# Vulnerability and stressors on the pathway to depression in a global cohort of young athletics (track and field) athletes

**DOI:** 10.1038/s41598-022-12145-0

**Published:** 2022-05-12

**Authors:** Toomas Timpka, Örjan Dahlström, Kristina Fagher, Paolo Emilio Adami, Christer Andersson, Jenny Jacobsson, Carl Göran Svedin, Stéphane Bermon

**Affiliations:** 1 Athletics Research Center, Linköping University, 581 83 Linköping, Sweden; 2 Department of Health, Medicine, and Caring Sciences, Linköping University, 581 83 Linköping, Sweden; 3 Department of Behavioural Sciences and Learning, Linköping University, 581 83 Linköping, Sweden; 4 Department of Health Sciences, Rehabilitation Medicine Research Group, Lund University, 221 00 Lund, Sweden; 5 World Athletics, Health and Science Department, 98007 Monte Carlo, Monaco; 6 University of Rome “Foro Italico”, 00135 Rome, Italy; 7 Department of Social Science, Marie Cederschiöld University, 100 61 Stockholm, Sweden; 8 LAMHESS, Université Côte d’Azur, 06205 Nice Cedex 03, France

**Keywords:** Diseases, Risk factors

## Abstract

This research set out to identify pathways from vulnerability and stressors to depression in a global population of young athletes. Retrospective data were collected at age 18–19 years from Athletics athletes (n = 1322) originating from Africa, Asia, Europe, Oceania, and the Americas. We hypothesised that sports-related and non-sports-related stressors in interaction with structural vulnerability instigate depression. Path modelling using Maximum likelihood estimation was employed for the data analysis. Depression caseness and predisposition were determined using the WHO-5 instrument. Thirty-six percent of the athletes (n = 480) returned complete data. Eighteen percent of the athletes reported lifetime physical abuse, while 11% reported sexual abuse. Forty-five percent of the athletes had recently sustained an injury. The prevalence of depression caseness was 5.6%. Pathways to depression caseness were observed from female sex (*p* = 0.037) and injury history (*p* = 0.035) and to predisposition for depression also through exposure to a patriarchal society (*p* = 0.046) and physical abuse (*p* < 0.001). We conclude that depression in a global population of young athletes was as prevalent as previously reported from general populations, and that universal mental health promotion in youth sports should include provision of equal opportunities for female and male participants, injury prevention, and interventions for abuse prevention and victim support.

## Introduction

Depression rates are increasing worldwide, making the disorder one of the dominant global health problems^1,2^. Stressful life events are present before symptom onset in about half of diagnosed cases^3^, and experience of such events has been suggested to characterize a subtype of the disorder^4^. A hypothesis is that stressors induce lasting depressed mood from young age through psychopathology such as dysregulation of the hypothalamic–pituitary–adrenal axis^5^. Participation in organised sports is a popular activity among young people worldwide with beneficial effects on health and social function^6^. However, stressors that may counteract these positive effects include the risk of sustaining injuries^7^ and demands from coaches and parents^8^. Regarding stressors outside sports, over half of all children and adolescents aged 2–17 years worldwide (N = 1 billion) are estimated to have experienced childhood abuse^9^.

While 20–30% of the general populations in Asia and Africa has experienced sexual abuse before adulthood, the prevalence in Europe and North America is about 10%^10^. The most common childhood abuse perpetrator is a family member^11^. One potential explanation of the difference in abuse prevalence between the continents is therefore the nature of family relations^12–14^. In collectivistic-patriarchal family settings, common in Asia and Africa^12,15^, social conventions restrict the personal freedom of women and young people; adolescent marriages and dowry are examples of arrangements that here contribute to life stress^16^. In India, for instance, on average 21 women died each day in 2015 due to dowry harassment^17^. The patterns of abuse in traditional patriarchal families is supported and reinforced by societal values and gender inequality that place females in subordinate positions to males^18^.

Athletics (track and field) is a global sport organized into 214 national federations^19^. National-level studies have revealed notable occurrences of abuse and mental health issues among adult Athletics athletes^20,21^. The diathesis-stress model^5,22^ assumes that depressive symptoms develop from an interaction between stress and an individual’s vulnerability, or diathesis. Some level of structural vulnerability is supposed to be present in everybody, with a threshold over which symptoms will appear. Exceeding this threshold depends on the interaction between vulnerability and the degree of adversity faced in life events^23^.

The aim of this study was to examine pathways from vulnerability and stressors to depression in a global population of young Athletics athletes. We hypothesised that sports-related and non-sports-related stressors in interaction with structural vulnerability instigate depression. The overall purpose of the research is to develop knowledge that can be used in establishment of universal mental health promotion strategies for application in youth sports.

## Results

Of 1322 eligible athletes, 480 (36%) returned complete data sets, 229 females (48%) and 251 males with a mean age of 18.7 years (range 17.1–19.8 years). There was no significant difference in response rate between female and male athletes (*p* = 0.82). The participation was lower among athletes from North America (27%) and Africa (27%), and higher in athletes from South America (70%) and Asia (46%) (*p* < 0.001).

Twenty-four percent of the athletes (45% females; 55% males) originated from patriarchal norms societies and 76% from non-patriarchal societies (48% females; 52% males). The amount of training hours per week were almost identical for females (M 14.8, SD 6.20, n = 220) and males (M 14.9, SD 6.20, n = 238).

### Depression prevalence

The mean WHO-5 score (predisposition to depression) was 75.0 (SD 15.4) (females 73.0 (SD 15.3); males 76.8 (SD 15.2). The prevalence of depression caseness (WHO-5 score < 50) among the study participants was 5.6% (girls 7.9%; boys 3.6%) (Table 1).

**Table 1:** Predisposition to depression (mean WHO-5 score) and depression caseness (WHO-5 score < 50) among participating adolescent Athletics athletes (n = 480) displayed by sex and society.

	Depression predisposition; WHO-5, *M* (*SD*), *n*_*tot*_			Depression caseness; *n*_*dep*_ (%)		
	Patriarchal society^a^	Non-patriarchal society^b^	All societies	Patriarchal society	Non-patriarchal society	All societies
Females	70.0 (17.0), 53	73.8 (14.8), 176	73.0 (15.3), 229	5 (9.4%)	13 (7.4%)	18 (7.9%)
Males	74.1 (17.6), 64	77.7 (14.3), 187	76.8 (15.2), 251	3 (4.7%)	6 (3.2%)	9 (3.6%)
Total	72.2 (17.4), 117	75.8 (14.7), 363	75.0 (15.4), 480	8 (6.8%)	19 (5.2%)	27 (5.6%)

*M* mean, *SD* standard deviation, *n*_*tot*_ number of athletes, *n*_*dep*_ number of athletes with caseness depression (WHO-5 < 50). 26 athletes excluded due to missing data on more than one WHO-5 item.

^a^Asia, Africa, ^b^Europe, the Americas, Oceania.

### One-year injury prevalence

The prevalence of having sustained an injury that caused absence from regular participation in sports during the past year among the participating athletes was 44.8% (females 41.4%; males 48.0%) (Table 2). The most common injury cause was overuse (91.8%). Most of the injuries (74.4%; 71.7% in females and 76.5% in males) caused more than 7 days of absence from athletics, and 42.2% (40.2% in females and 43.7% in males) more than 21 days of absence.

**Table 2:** Prevalence of pre-competitions injury (≥ 3 week stime-loss) in numbers (percent) among participating adolescent Athletics athletes (n = 480) displayed by injury type, sex, and category of society.

	Patriarchal society^a^			Non-patriarchal society^b^			All societies		
	Females	Males	Total	Females	Males	Total	Females	Males	Total
Injury	19 (36.5%)	37 (57.8%)	56 (48.3%)	75 (42.9%)	82 (44.6%)	157 (43.7%)	94 (41.4%)	119 (48%)	213 (44.8%)
Traumatic	1 (1.9%)	5 (7.8%)	6 (5.2%)	17 (9.7%)	16 (8.7%)	33 (9.2%)	18 (7.9%)	21 (8.5%)	39 (8.2%)
Overuse sudden	10 (19.2%)	21 (32.8%)	31 (26.7%)	38 (21.7%)	42 (22.8%)	80 (22.3%)	48 (21.1%)	63 (25.4%)	111 (23.4%)
Gradual	8 (15.4%)	11 (17.2%)	19 (16.4%)	19 (10.9%)	23 (12.5%)	42 (11.7%)	27 (11.9%)	34 (13.7%)	61 (12.8%)
N/A	0 (0%)	0 (0%)	0 (0%)	1 (0.6%)	1 (0.5%)	2 (0.6%)	1 (0.4%)	1 (0.4%)	2 (0.4%)
n	53	64	117	176	187	363	229	251	480

^a^Asia, Africa, ^b^Europe, the Americas, Oceania.

### Lifetime sexual and physical abuse prevalence

About one quarter of the athletes (23.8%) reported lifetime abuse experiences; about the same proportion in females (24.0%) and males (23.5%) (Table 3). Eighteen percent of the athletes reported having sustained lifetime physical abuse; the same proportion (18.3%) in females and males. Regarding lifetime sexual abuse, 11% of the participating athletes reported victimization; about the same proportion in females (9.6%) and males (12.4%).

**Table 3:** Abuse experiences (physical, sexual) among participating adolescent Athletics athletes (n = 480) displayed by society and sex.

	Patriarchal society^a^			Non-patriarchal society^b^			All societies		
	Females	Males	Total	Females	Males	Total	Females	Males	Total
**Abuse type**									
Physical abuse	13 (24.5%)	17 (26.6%)	30 (25.6%)	29 (16.5%)	29 (15.5%)	58 (16.0%)	42 (18.3%)	46 (18.3%)	88 (18.3%)
Sexual abuse	6 (11.3%)	8 (12.5%)	14 (12.0%)	16 (9.1%)	23 (12.3%)	39 (10.7%)	22 (9.6%)	31 (12.4%)	53 (11.0%)
Any abuse	16 (30.2%)	22 (34.4%)	38 (32.5%)	39 (22.2%)	37 (19.8%)	76 (20.9%)	55 (24.0%)	59 (23.5%)	114 (23.8%)
n	53	64	117	176	187	363	229	251	480

^a^Asia, Africa, ^b^Europe, the Americas, Oceania.

### Pathways from vulnerability and stressors to depression

**Figure 1:**
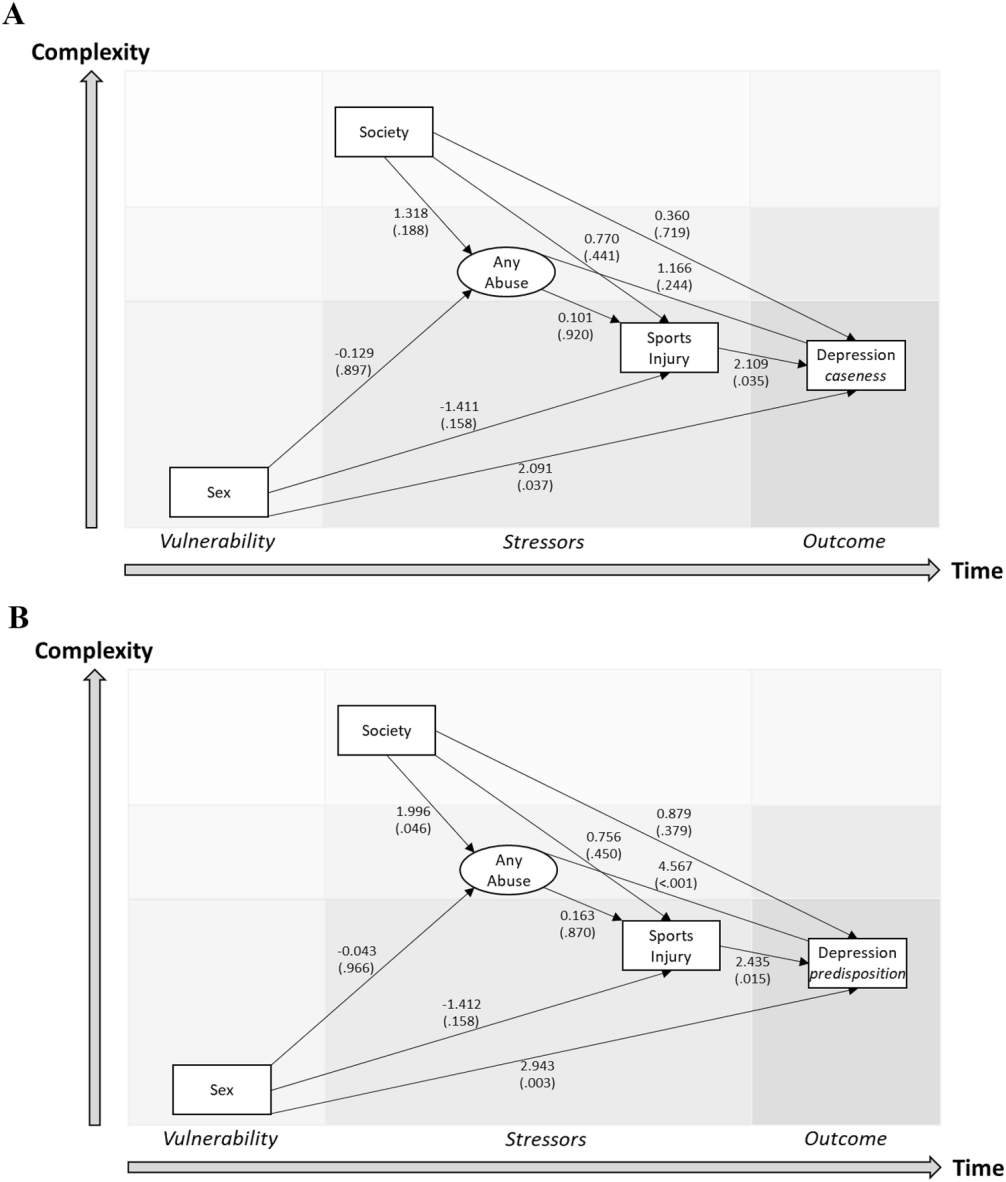
Diathesis-stress models of depression etiology in young sportspersons where abuse is represented by any lifetime abuse (either lifetime sexual or physical abuse). Models of (**A**) depression caseness (WHO-5 score < 50), and (**B**) predisposition to depression (crude WHO-5 score) are displayed.

The pathway model to depression caseness combing any abuse experience included only direct paths from vulnerability (female sex) (*p* = 0.037) and recent injury event (*p* = 0.035) (Fig. 1A). The corresponding model of predisposition to depression also contained direct paths from vulnerability (female sex) (*p* = 0.003) and recent injury event (*p* = 0.015), and an indirect path from patriarchal society (*p* = 0.046) and lifetime abuse experience (*p* < 0.001) (Fig. 1B).

**Figure 2:**
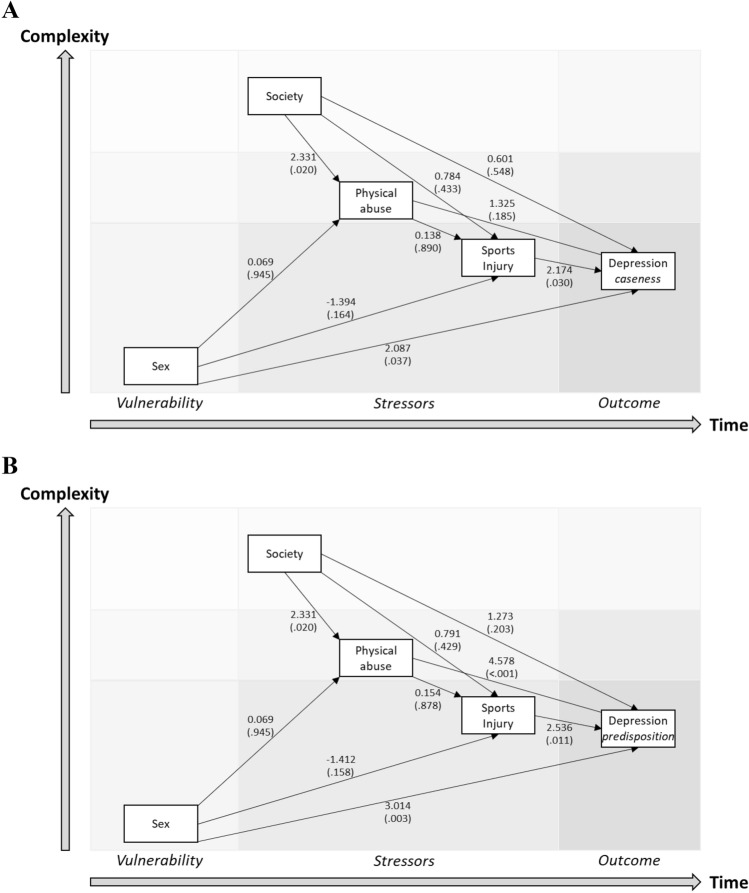
Diathesis-stress models of depression etiology in young sportspersons where abuse is only represented by lifetime physical abuse. Models of (**A**) depression caseness (WHO-5 score < 50), and (**B**) predisposition to depression (crude WHO-5 score) are displayed.

The pathway model to depression caseness including only physical abuse contained two direct paths: from female sex (*p* = 0.037) and recent injury event (*p* = 0.030) (Fig. 2A). The corresponding model of predisposition for depression contained direct paths from female sex (*p* = 0.003) and injury event (*p* = 0.011) but also an indirect path from patriarchal society (*p* = 0.020) and lifetime abuse experience (*p* < 0.001) (Fig. 2B).

Lifetime sexual abuse was not associated with any of the other investigated stressors, or any depression outcome. The pathway model to depression caseness including only sexual abuse contained direct links from female sex (*p* = 0.039) and injury event (*p* = 0.030). Also, the model of predisposition for depression displayed direct paths from female sex (*p* = 0.003) and recent injury event (*p* = 0.012).

## Discussion

The aim of this research was to examine pathways from vulnerability and stressors to depression in a global population of young Athletics athletes. We found pathways to depression caseness from female sex and recent injury event and to predisposition for depression also through exposure to a patriarchal society and physical abuse. In line with a recent meta-analysis^24^, the observed depression caseness and predisposition levels among the young sportspersons were similar to those reported from general populations^25^.

The observation that females were more vulnerable for depression is in accordance with previous studies^26^. Narrowing of sex differences in depression prevalence has during the recent decades been related to universal changes in gender role traditionality^27^. Upturns in female opportunities in domains such as employment and birth control increase gender role equality and promote improvements in female mental health by reducing the background structural vulnerability of women^28^. Participation by women in high-level sport has increased during the recent decades, as have professional sports opportunities for female athletes. In 1900, 2.2% of the competitors in the Olympic Games were female; in 1952 the proportion was 10.5%, in 2000 it was 38.2% and in 2021 it was 48.8%^29^. To further reduce the background structural vulnerability to depression among females, our findings imply that universal mental health promotion in youth sports should include continued efforts to provide equal opportunities for female and male participants.

Our finding that injury events trigger depression in young athletes is in concordance with previous studies^8^. Several mechanisms effectuating the link from injury events to depression in sportspersons are plausible. The approach to injury in athletes has been presented as a process, influenced by situational factors, that begins pre-injury with the athlete’s possession of coping strategies and continues with cognitive appraisal through the injury phase and rehabilitation^30^. Young competitive athletes may experience injuries more stressful than their adult peers because they seldom can manage injury symptoms by themselves^31^ and often simultaneously must deal with critical career transitions^32^. Adolescents are also known to face larger structural barriers than children and adults in accessing healthcare services^33^, which has particular implications for young athletes. Consequently, it is reasonable to assume that provision of improved access to injury prevention and medical support would reduce stress and reduce the risk of depression among competitive young athletes.

Regarding predisposition for depression in young athletes, we found a novel path from collectivistic-patriarchal societies through physical abuse to depression. There is robust evidence of meaningful associations between exposure to childhood physical abuse and increased likelihood of depression later in life^34^. It is also known that the most common perpetrator of childhood physical abuse is a family member^11^. In this study among young sportspersons, we found that a lifetime history of physical abuse was more frequent in participants from Asia and Africa. Even though individual-level perpetrator data were not analyzed, families in Asian and African cultures are more likely be structured in collectivistic-patriarchal systems^12–14^. These family systems have been criticized for using force and violence to solve conflicts and maintain their influence at the expense of women and young people^15,35^. With industrialization and urbanization, the collectivistic support functions of patriarchal family systems have progressively been transferred to welfare state agencies and legislators through, e.g. through introduction of social insurance schemes and laws prohibiting corporal punishment of children^36,37^. Therefore, the mental health consequences of physical abuse observed in this study warrant consideration of culturally adjusted interventions for abuse prevention and victim support among young athletes that are implemented in collaborations between sports organisations, public health agencies, and mental health services.

We observed no effect of exposure to sexual abuse on depression. Childhood sexual abuse has been identified as a predictor of depression over the life course, while depression has been linked with other medical and behavioral health issues^38^. An explanation for the observation that no effect on depression was found is that the association works over a prolonged period and is mediated by intermediate conditions^39^. In other words, it is possible that effects of childhood sexual abuse on depression in athletes are triggered through conditions such as injuries or relationship issues, and do not show before adulthood. Moreover, contrary to previous findings in non-athletes^40^, we observed similar prevalence of lifetime sexual abuse in females and males. One interpretation of this finding is that young male high-level athletes are more exposed to high-risk environments than their non-athlete peers^41^. It also may be that the true sexual abuse prevalence in boys is higher in non-Western settings, while most previous studies have been performed in Western contexts^10^. However, we also found depression caseness and predisposition to be higher in females. A possible explanation of this inconsistency is that the effects of sexual abuse differ between males and females and that the effects in males show as maladaptive behavior rather than mood disorders^42^. Other explanations of the inconsistency include sampling bias^43^, and underreporting of sexual abuse among males in general population studies^44^. Nonetheless, the high proportion of males reporting a history of sexual abuse in this global population of young athletes is noteworthy and warrants further research.

This is the first study to examine pathways to depression in a global population of young athletes. Despite reports of increasing rates of depression in young people worldwide, there are few studies comparing determinants in global regions using a common methodology. Other strengths of the study include that the WHO-5 instrument is validated in all languages used for the survey and that the items do not ask for symptoms of depression. Athletes may fear to deal with the stigma associated with mental illness and therefore be reluctant to report symptoms of mental health disorders^45^. The proposed cut-off score of < 50 on the WHO-5 was used to define depression caseness, corresponding to a screening diagnosis of depression^25^. The caseness proportions should only be compared with results obtained using the same or corresponding definitions. Regarding limitations, additional vulnerabilities and stressors could have been analysed, e.g. demands from coaches and parents^8^. However, this would have increased the cognitive burden of responding to the survey with potential negative consequences for response rates. A specific limitation is that, based on expected small numbers of LGBTQ athletes (56 LGBTQ persons out of 11,238 athletes in the 2016 Summer Olympics^46^, we decided to only ask for male and female sex. With the rising proportion of LGBTQ persons^47^, this group warrants more attention in future research. Moreover, the participating young athletes had qualified for the World Championships, implying that they from a sports perspective were better off than many of their peers and that the risk of selection bias should be considered when interpreting the results. Even though the overall study participation was satisfactory, lower response rates were observed among African and North American athletes. Nonetheless, no indication was found to suggest that the results should be meaningfully skewed. Possible explanations of the non-response include that these subgroups of African athletes were not familiar with any of the study languages and that the North American team stayed at a camp outside the designated championship area and was not readily reached for study invitation. Finally, despite extensive population movements the recent decades, geographical differences in collectivistic values and motivations between world regions remain^48^ that support the categorisation into patriarchal and non-patriarchal societies used in this study.

In this the first study of pathways to depression in a global population of young Athletics athletes, we found paths to depression caseness from female sex and recent injury history, and to predisposition for depression from exposure to a patriarchal society and physical abuse. The results imply that universal mental health promotion strategies in youth sports should include provision of equal opportunities for female and male participants, injury prevention, and interventions for abuse prevention and victim support.

## Methods

The study was based a retrospective cross-sectional design. The design followed the Helsinki declaration of ethical principles for medical research. All subjects provided informed consent to participate in the study. Ethical approval of the study design was obtained from the research ethics board of Linköping University (Dnr. 2018-222-31).

### Theoretical model

**Figure 3:**
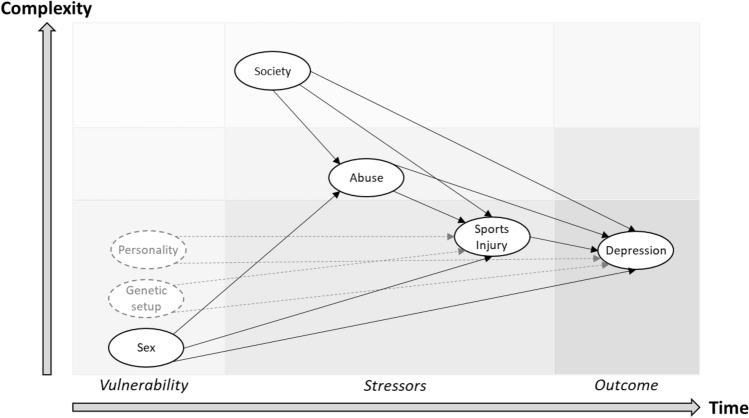
Diathesis-stress model of pathways from structural vulnerability and stressors to depression in young sportspersons (adapted from Ref.^23^).

A diathesis-stress model^22^ was adapted for structuring of the data collection and analyses (Fig. 3). It was assumed that female sex constitutes a structural vulnerability with regards to depression^26^. Vulnerability with regards to genetic background and personality was not included in the present model. The sports-related stressor included in the model was having recently suffered an injury, while the life stressors were being exposed to patriarchal family norms, lifetime history of physical abuse, and lifetime history of sexual abuse.

### Study population

The primary study population consisted of athletes registered for the World Athletics Under 20 World Championships in Tampere 2018 and belonged to a team that mastered at least one of the World Athletics official languages. National teams unfamiliar with these languages or with a restricted team communication policy were excluded.

### Data collection

Data were collected using a self-administered web-based preparticipation health questionnaire (PPHQ) involving a code-based procedure for athlete anonymization. The PPHQ was translated into the seven study languages (English, Spanish, French, Portuguese, Amharic, Chinese, and Japanese). The eligible athletes were first informed about the study before the championships through their team managers. At dedicated stalls at the stadium and training arenas, the athletes were thereafter personally invited to complete the PPHQ through hand-out of leaflets containing detailed study information and the website link.

The PPHQ collected information regarding athlete sex, age, and country. Data on depression was collected using the WHO-5 instrument^49^. A systematic review based on 213 papers reported WHO-5 to have high clinimetric validity and assessed it as a sensitive and specific screening tool for depression with high applicability across study fields^25^. An early indicator of depression in young people is reduced well-being^25,50^. The WHO-5 employs positively formulated statements about health status instead of asking for symptoms of disease. Young competitive athletes were considered to perceive questions about depressive mood as less relevant and be more inclined to report on their well-being. The WHO-5 consists of five positively worded items that reflect the presence or absence of well-being rather than depressive symptomatology. The athletes were asked to report the presence of these positive feelings in the last 2 weeks on a 6-point scale ranging from all of the time (5 points) to at no time (0 point). Each score is multiplied with 4 rendering a total score ranging from 0 to 100. Predisposition to depression was measured by level of well-being as measured by the crude WHO-5 score and depression caseness was defined as scoring < 50 on WHO-5^25^.

Sports injury data were collected by asking whether the athlete had suffered an injury that caused absence from regular participation in sports during the past year, henceforth referred to as a recent injury event. Structured follow-up questions were asked about injury cause and length of absence from sports.

Sexual abuse was defined as any sexual interaction involving physical contact with person(s) of any age that was perpetrated against the victim’s will, without consent or in an aggressive, exploitative, manipulative, or threatening manner. Precisely, the variable used to collect data on sexual abuse was derived from the statement and questions originally developed by Mossige^51^. Physical abuse was defined as being deliberately hurt by an adult person causing injuries such as bruises, broken bones, burns or cuts^52^. The specific questions asking for lifetime physical and sexual abuse experiences have been described previously^53,54^.

### Data analyses

Before the analysis, nations and continents were recoded by prevailing family system into patriarchal norms (Africa/Asia) and non-patriarchal norms (the Americas/Europe/Oceania) societies, based on the definition that a patriarchal society is a social system in which power is held by men, through cultural norms and customs that favor men and withhold opportunity from women)^12,14^. Depression caseness and predisposition to depression and were used as endpoints for path modelling analyses. When a determinant affects an outcome, it may be of interest to explain the underlying mechanism. Pathway analyses approach this by identifying intermediate variables that are also affected by the determinant, and in turn affect the outcome. The effects of the determinant that pass through these mediators are termed “indirect” effects. Here pathways from vulnerability and stressors to depression were examined based on the diathesis-stress model (Fig. 1). The determinants examined were sex (female, male), sport injury (causing more than 7 days of absence from athletics) the previous year (yes, no), life-time sexual abuse (yes, no), life-time physical abuse (yes, no), and society of origin (patriarchal/non-patriarchal norms). WHO-5 crude scores were used for analyses of predisposition of depression and WHO-5 scores < 50 (yes/no) were used as cut-off level for depression caseness^25^. Predisposition to depression and depression caseness were analyzed separately. Significance was tested for paths between different types of abuse [sexual abuse, physical abuse, any abuse (latent variable combing sexual and physical abuse)] and depression taking into consideration the influence of sex. Non-significant paths were stepwise removed until only significant paths remained. Path modeling was performed using Mplus software (8th edition) using Maximum likelihood estimation with robust standard errors.

## Data Availability

The datasets generated and/or analysed during the current study are not publicly available due Swedish legislation regarding research ethics but are available from the corresponding author on reasonable request.
